# Enhancing Person Re-Identification through Attention-Driven Global Features and Angular Loss Optimization

**DOI:** 10.3390/e26060436

**Published:** 2024-05-21

**Authors:** Yihan Bi, Rong Wang, Qianli Zhou, Ronghui Lin, Mingjie Wang

**Affiliations:** 1 School of Information and Cyber Security, People’s Public Security University of China, Beijing 100038, China; rcbyh2023@126.com (Y.B.);; 2 Key Laboratory of Security Prevention Technology and Risk Assessment of Ministry of Public Security, Beijing 100038, China; 3 Beijing Public Security Bureau, Beijing 100038, China

**Keywords:** person re-identification, global feature learning, attention mechanism, classification loss optimization

## Abstract

To address challenges related to the inadequate representation and inaccurate discrimination of pedestrian attributes, we propose a novel method for person re-identification, which leverages global feature learning and classification optimization. Specifically, this approach integrates a Normalization-based Channel Attention Module into the fundamental ResNet50 backbone, utilizing a scaling factor to prioritize and enhance key pedestrian feature information. Furthermore, dynamic activation functions are employed to adaptively modulate the parameters of ReLU based on the input convolutional feature maps, thereby bolstering the nonlinear expression capabilities of the network model. By incorporating Arcface loss into the cross-entropy loss, the supervised model is trained to learn pedestrian features that exhibit significant inter-class variance while maintaining tight intra-class coherence. The evaluation of the enhanced model on two popular datasets, Market1501 and DukeMTMC-ReID, reveals improvements in Rank-1 accuracy by 1.28% and 1.4%, respectively, along with corresponding gains in the mean average precision (mAP) of 1.93% and 1.84%. These findings indicate that the proposed model is capable of extracting more robust pedestrian features, enhancing feature discriminability, and ultimately achieving superior recognition accuracy.

## 1. Introduction

Person re-identification tasks aim to retrieve and match specific pedestrians from multiple nonoverlapping cameras [1] and have attracted widespread attention in academia in recent years, becoming a research hotspot. This technology can be combined with facial recognition, pedestrian detection, etc., to promote the development of intelligent security fields such as suspect tracking.

Traditional person re-identification consists of two sub-tasks, feature extraction and similarity measurement, which cannot achieve end-to-end processing from raw data to results. It is inefficient and time-consuming and generally faces the problem of low accuracy. With the rise of deep learning, person re-identification integrates feature extraction and similarity measurements into a unified framework. By using convolutional neural networks (CNNs) to extract high-level features of pedestrians and designing loss functions to control intra-class and inter-class distances, the performance of person re-identification is greatly improved.

The challenge of a person re-identification task lies in adequately expressing and discriminating pedestrian features. In practical situations, various factors, including camera angles, lighting conditions, occlusions, and intricate backgrounds, hinder the performance of person re-identification methods. These factors lead to an insufficient representation of pedestrian features, compromised discriminatory power, and reduced recognition accuracy [2]. Consequently, the extraction of more distinctive pedestrian features and the adoption of effective similarity measurement methods have become crucial for enhancing inter-class differences and minimizing intra-class variations in person re-identification.

In response to this situation, we optimized the person re-identification method based on global feature learning in both feature extraction and similarity measurement. We propose a person re-identification method based on global feature learning and classification optimization which fully extracts key pedestrian features, makes the network model output more discriminative regarding pedestrian features, and improves the accuracy of person re-identification. This method is based on the backbone network ResNet50, and our main contributions can be summarized as follows:

- Incorporating the Normalization-based Channel Attention Module [3] into the residual module of the backbone network ResNet50 and using scaling factors to weight and strengthen key pedestrian feature information so that the network model selectively enhances key features and suppresses useless features;
- Using the dynamic activation function Dynamic ReLU [4], which only shares spatial dimensions, as the activation layer of the backbone network, the parameters of ReLU are dynamically adjusted based on the input convolutional feature maps to enhance the nonlinear expression ability of the network model for pedestrian features;
- Introducing Arcface loss [5] into cross-entropy loss, the supervised network model learns pedestrian features with large inter-class distance and small intra-class distance, making the network model output more discriminative pedestrian features.

## 2. Related Work

### 2.1. Overview of Person Re-Identification

A diverse array of person re-identification methods have been put forth, encompassing methods rooted in feature learning, metric learning [2], and those utilizing generative adversarial networks [6] (GANs). Depending on the varying representations of pedestrian features, feature learning-based methods can broadly be categorized into those focused on global feature extraction, those emphasizing local feature analysis, and those leveraging auxiliary feature learning [7].

Methods of person re-identification based on global feature learning involve representing an entire image as a single feature vector, followed by the measurement of image similarity. Wang et al. [8] proposed a person re-identification framework that combines single-image feature matching and cross-image feature matching and utilized convolutional neural networks (CNNs) to extract pedestrian features. Zhang et al. [9] proposed a person re-identification method based on global features which utilizes a multi-receptive field fusion module and an attention module to fully obtain pedestrian contextual information, as well as a multi-branched network structure to fuse features of different depths to predict pedestrian identity.

Methods of person re-identification based on local feature learning represent an entire image as a set of local features and then measure the similarity of the image. This can be achieved through methods such as dividing pedestrian components or simple vertical region partitioning. Zhao et al. [10] proposed a multi-stage feature decomposition and fusion method for pedestrian re-identification guided by human body regions, taking into account human body structural information. Wang et al. [11] proposed a pose-driven local feature alignment method for person re-identification, which uses a pose encoder to guide the network in extracting visible features of pedestrians and uses a pedestrian component alignment module to extract local features of pedestrians and align them to reduce interference from non-pedestrian features.

Methods of person re-identification based on auxiliary feature learning enhance the expression of pedestrian features through auxiliary information such as instance attributes and identity attributes. Lin et al. [12] optimized recognition performance on large-scale data by utilizing complementary information learned from attribute labels.

### 2.2. Methods Based on Global Feature Learning

In 2016, Zheng et al. [13] proposed the IDE model, which regarded person re-identification as a classification task and became a baseline model for many subsequent works. For example, in terms of feature extraction, Sun et al. [7] addressed the issue of insufficient pedestrian feature expression by embedding the channel attention mechanism’s SE module into the backbone network, enabling the network model to output more representative pedestrian expression features. Du et al. [14] introduced a spatial attention mechanism to improve the global feature extraction network of the person re-identification algorithm with topological relation occlusion, taking into account the information interference problems in scenes such as occlusion and complex backgrounds. Lin et al. [15] proposed a method called the Dual Descriptor Feature Enhancement (DDFE) network, which uses two independent sub-networks to extract descriptors from the same pedestrian and combine them to create a comprehensive multi-view representation, significantly improving person re-identification performance. In terms of similarity measurement, commonly used loss measures include contrast loss [16], triplet loss [17], quadruple loss [18], etc. Zhang et al. [19] improved the original triplet loss and optimized the intra-class distance.

Among the methods mentioned above, methods that focus on innovation in feature extraction [7,13,14,15] can easily cause problems such as a network model having insufficient generalization ability and misfitting deviations in the training data. Methods that focus on improving the loss function [16,17,18,19] can easily cause problems such as disappearing or exploding gradients and difficulty in capturing complex nonlinear relationships in feature spaces. In contrast, combining feature extraction and similarity measurement and selecting appropriate methods to make them complement each other can help effectively deal with the above problems and optimize the performance of the network model globally and harmoniously.

In recent years, in order to improve the accuracy of person re-identification on datasets, a large amount of work has adopted a combination of global feature learning and local feature learning methods. Wang et al. [20] designed and introduced PAN modules into the global feature branch and designed local feature branches to enable the model to better learn fine-grained features of local regions while preserving the correlation information between regions. Zhang et al. [21] proposed a person re-identification method that utilizes feature fusion and multi-scale information. After extracting the global feature map of a human body image, the global feature map is horizontally segmented to obtain local features. By combining adaptive spatial feature alignment, local feature fusion, and multi-scale feature extraction, pedestrian features are obtained.

Person re-identification methods that obtain local features of pedestrians through horizontal segmentation usually involve multiple branches, which increases the complexity of the network structure, and rough partitioning can easily cause misalignment problems between corresponding components. In contrast, person re-identification methods based on global feature learning have a clear design and a simpler network model process, which has great research significance.

## 3. Methods

In this section, the framework of the network model proposed in this paper is introduced in Section 3.1, followed by a detailed explanation of three specific optimizations in feature extraction and similarity measurement in the proposed method from Section 3.2, Section 3.3 and Section 3.4.

### 3.1. Framework of the Proposed Method

The process of person re-identification methods based on global feature learning is roughly as follows: After extracting features from the backbone network, a global average pool (GAP) is used to obtain a global feature vector, which is then processed through a fully connected layer to obtain low-dimensional output features. Then, the triplet loss and cross-entropy loss are used as the measurement loss and classification loss for the similarity measurement, respectively. This type of method has a simple structure but usually lower accuracy.

**Figure 1:**
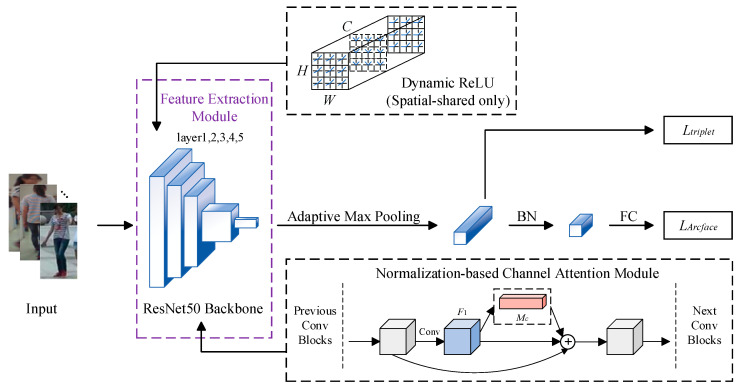
Overall architecture of the proposed method.

On this basis, we proposed a novel person re-identification method based on global feature learning. For the structural framework of the network model, we removed the global average pooling layer and fully connected layer at the end of the backbone network ResNet50 and added an adaptive maximum pooling layer to output 2048 × 4 × 1 feature vectors. For both feature extraction and similarity measurement, we made the following 3 specific optimizations. The network structure and overall process of our method are shown in Figure 1. Among them, BN represents batch normalization [22] and FC represents a fully connected layer. $L_{\mathrm{triplet}}$ represents triplet loss, and $L_{\mathrm{Arcface}}$ represents cross-entropy loss after introducing Arcface loss; they are used as the measurement loss and classification loss for the similarity measurement after obtaining output features. In the Dynamic ReLU (spatial-shared only), H represents the height of the feature graph, W represents the width of the feature graph, and C represents the number of channels in the feature graph. In the Normalization-based Channel Attention Module, $F_{1}$ represents the input features put into the attention module and $M_{C}$ represents the corresponding output feature.

### 3.2. Normalization-Based Channel Attention Module

An attention mechanism can help neural network models suppress less significant features in channels, reduce interference caused by irrelevant information, and thus improve discriminative power regarding pedestrian features. We added the Normalization-based Channel Attention Module as a lightweight attention mechanism in the channel dimension using a scaling factor for batch normalization [22] (BN), which uses the standard deviation to represent the importance of weights, avoiding the addition of fully connected and convolutional layers present in other attention models such as Squeeze and Excitation [23] (SE) modules and convolutional block attention modules [24] (CBAMs), which makes computation more efficient while maintaining performance.

**Figure 2:**
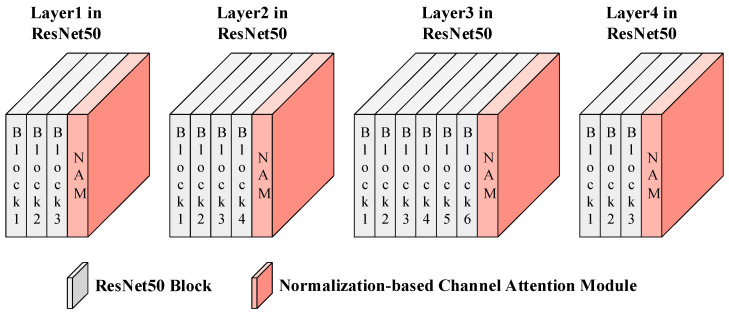
Normalization-based Channel Attention Module in the proposed method.

For the backbone network ResNet50, the Normalization-based Channel Attention Module was embedded after the last block from Layer 1 to Layer 4, with each block embedding a Normalization-based Channel Attention Module, as shown in Figure 2. Among them, NAM is short for the Normalization-based Channel Attention Module, as is the case below.

The Normalization-based Channel Attention Module utilizes the scaling factor, γ, of batch normalization [22] to measure the variance of each channel and reflect the magnitude of the changes in each channel, thereby indicating the importance of that channel. The role of the scaling factor in BN is shown in Equation (1). In the equation, $B_{\mathrm{in}}$ and $B_{\mathrm{out}}$ represent the input and output of *BN*, respectively.

(1) $$B_{out}=BN\left(B_{in}\right)=\gamma \frac{B_{in}-\mu_{B}}{\sqrt{\sigma_{B}^{2}+\varepsilon }}+\beta$$

In the equation, $\mu_{B}$ and $\sigma_{B}$ are the mean and standard deviation of a small batch B, respectively, and γ and β are trainable affine transformation parameters (scaling and shifting). After obtaining the scaling factor, γ, for each channel, the weight, $W_{\gamma },$ for each channel can be calculated using Equation (2).

(2) $$W_{\gamma }=\frac{\gamma_{i}}{\sum_{j=0}\gamma_{j}}$$

After calculating the weight, $W_{\gamma },$ of each channel, for input feature $F_{1}$, we can obtain the corresponding output feature, $M_{C},$ through Equation (3).

(3) $$M_{C}=sigmoid\left(W_{\gamma }\left(BN\left(F_{1}\right)\right)\right)$$

**Figure 3:**
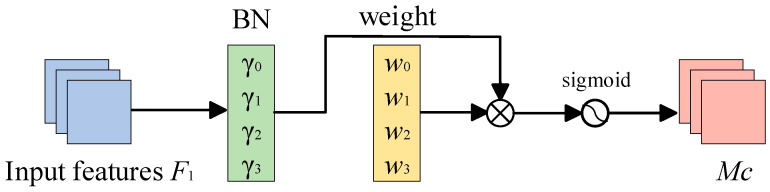
Specific workflow of the NAM.

The specific workflow of the Normalization-based Channel Attention Module is shown in Figure 3.

**Figure 4:**
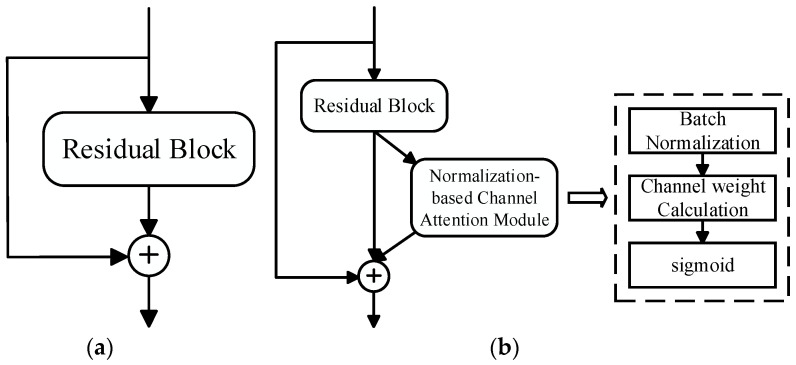
Residual structure of NAM-ResNet. (**a**) ResNet; (**b**) NAM-ResNet.

We incorporated the Normalization-based Channel Attention Module into the residual module of the backbone network ResNet50, which makes the pedestrian expression features extracted by the proposed person re-identification method more representative and generalized. Specifically, firstly, after the input was processed by the residual block, we used the Normalization-based Channel Attention Module to carry out the weighted adjustment of the feature channels, that is, to enhance the useful feature channels and weaken the redundant feature channels. Secondly, we added the basic feature information processed by the residual block to the key detail feature information weighted by the attention mechanism, aiming to improve the generalization and robustness of the model through a feature fusion strategy. Finally, we connected the input of the residual block to a deeper network layer through Identity Mapping [25], which adds neither extra parameters nor computational complexity. The residual structure of the network model after embedding the Normalization-based Channel Attention Module is shown in Figure 4.

### 3.3. Dynamic Activation Function

**Figure 5:**
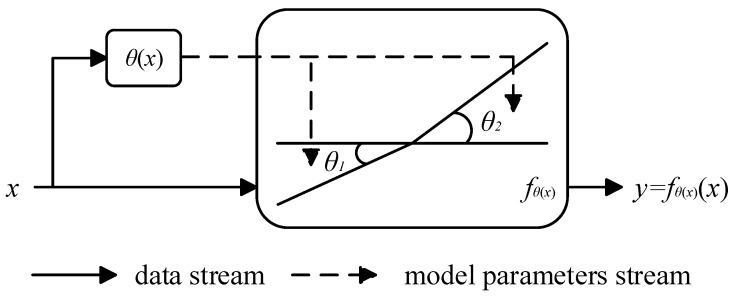
Schematic diagram of dynamic activation function.

For network models, the activation function affects their ability to express nonlinear modeling of input features. Compared to ReLU [26], Leaky ReLU [27], PreLU [28], etc., Dynamic ReLU can better capture the nonlinear expression of input features, mainly because this function can dynamically adjust the parameters of the activation function based on input features. As shown in Figure 5, the dynamic activation function is a piecewise function whose core hyperfunction, θ(x), is a function that dynamically adjusts the parameters of the activation function according to different inputs, and parameters $\theta_{1}$ and $\theta_{2}$ are obtained by the hyperfunction θ(x) on the input x. The hyperfunction θ(x) automatically adapts to the activation function by integrating the characteristics of various dimensions of the input, which can significantly improve the expressive ability of the network with a small amount of additional computation.

**Figure 6:**
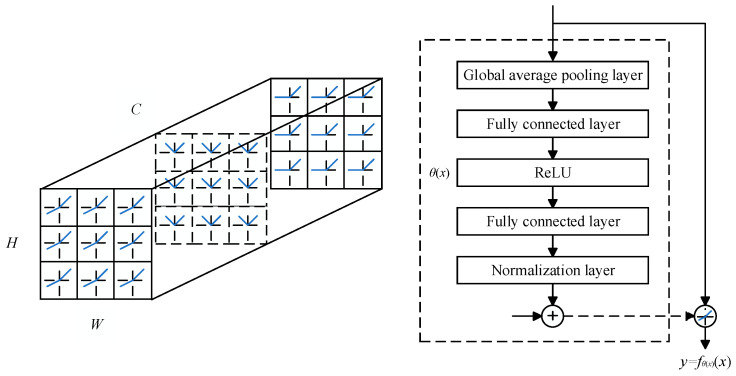
Dynamic ReLU (spatial-shared only).

In the dynamic activation function, the implementation of the hyperfunction θ(x) is achieved using the Squeeze and Excitation [23] (SE) module. As shown in Figure 6, for the input features with a size of C×W×H, the global average pooling layer is first used for compression, then two fully connected layers (including ReLU) are used for processing, and finally the output result is constrained between (−1,1) through the normalization layer, 2σ(x) − 1, where σ(x) is the sigmoid function.

There are three different forms of dynamic activation functions, specifically manifested as differences in sharing mechanisms in the spatial location and channel dimensions. We used the dynamic activation function, which only shares spatial positions, as the activation layer of the backbone network to enhance the ability of the network model to express pedestrian features.

### 3.4. Arcface Loss

In the context of feature learning, a suitable loss function plays a pivotal role in enabling the model to effectively extract valuable information from the available data. Consequently, this enhances the discriminative capabilities of the network model in extracting relevant features, thereby improving its overall performance.

The cross-entropy loss is widely used in image classification tasks. It calculates the probability of the extracted feature vectors belonging to each category through the softmax function and then calculates the loss. The network model is optimized by minimizing the difference between the true probability distribution and the predicted probability distribution. For the i-th learned feature $f_{i}$, the cross-entropy loss is represented as Equation (4). In this equation, N represents the number of images in a batch, $W_{k}$ represents the weight vector of the corresponding category, and C represents the number of categories of pedestrians in the training set.

(4) $$L1=-\frac{1}{N}\sum_{i=1}^{N}\log\frac{e^{W_{\left(y_{i}\right)}^{T}f_{i}}}{\sum_{k=1}^{C}e^{W_{k}^{T}f_{i}}}$$

The cross-entropy loss function makes pedestrian images close to their respective categories, achieving classification results. However, the cross-entropy function only focuses on the accuracy of predicting the probability of correct labels while ignoring the differences in other incorrect labels, which leads to scattered pedestrian features being learned by the network model. To overcome the aforementioned shortcomings of cross-entropy loss, we introduce Arcface loss [5] into the supervised network model, which learns pedestrian features with large inter-class distances and small intra-class distances, thereby making the network model output more discriminative in terms of pedestrian features. We implemented the following three steps to improve the cross-entropy loss by increasing the inter-class distance while reducing the intra-class distance in the angle space.

The initial step was to standardize the feature vectors, making sure that the module length was one. This standardization ensures consistency between the model weights and the distribution of feature vectors, ensuring that the distance measurement between samples solely relies on their angles. Subsequently, the introduction of the additive angle interval, margin (*m*), defined the classification boundary between positive and negative samples. By establishing limits on the maximum angle between similar samples and the minimum angle between dissimilar samples, we narrowed the angle range for each classification, significantly enhancing the distance between classes. Lastly, we added the scaling factor, scale (*s*), which acts as the normalized coefficient for the feature vector. When multiplied by the embedded dimension, it facilitates a more rapid training process for the model and accelerates the convergence of the loss function.

The cross-entropy loss after introducing the Arcface loss is shown in Equation (5). In this equation, *m* represents the additive angle margin, and *s* is the scaling factor scale.

(5) $$L2=-\frac{1}{N}\sum_{i=1}^{N}\log\frac{e^{s\left(\cos\left(\theta_{y_{i}}+m\right)\right)}}{e^{s\left(\cos\left(\theta_{y_{i}}+m\right)\right)}+\sum_{k=1,k\neq y_{i}}^{C}e^{s\left(\cos\theta_{k}\right)}}$$

## 4. Experiment and Analysis

### 4.1. Datasets and Evaluation Metric

**Figure 7:**
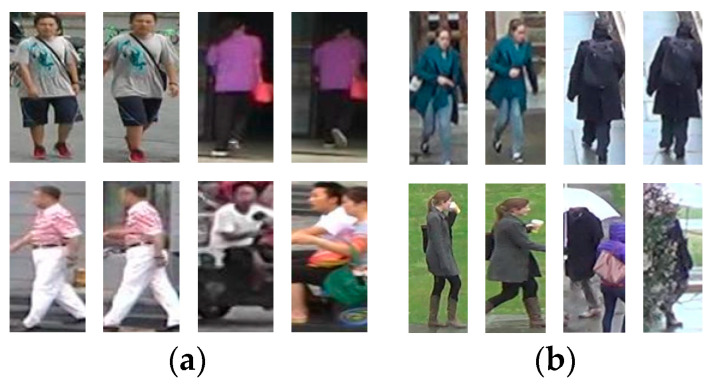
Image examples of the two datasets. (**a**) Market1501; (**b**) DukeMTMC-ReID.

We conducted experiments on the proposed person re-identification method on two mainstream datasets, Market1501 [29] and DukeMTMC-ReID [30], as shown in Table 1. The Market1501 dataset contains 1501 entity objects and 32,688 detected pedestrian rectangular boxes. Among them, the training set contains 751 entities and 12,936 images, while the test set contains 750 entities and 19,732 images; The DukeMTMC-ReID dataset contains 1404 entity objects and 36,411 pedestrian images (including 2228 query set images). The training set contains 702 entities and 16,522 images, while the test set contains 702 entities and 17,661 images. Image examples of the two datasets are shown in Figure 7. Among them, we selected eight images belonging to four identities (IDs) from each dataset, including four types of examples showing the front, back, side, and occlusions of pedestrians.

We evaluated the performance of the proposed person re-identification network model using the mean average precision (mAP) and cumulative match characteristic (CMC) as performance evaluation metrics. The CMC curve shows the probability of a correct match as the ranking of the query results increases. Here, Rank-m represents the hit rate of the search target in the first m bits of the sorted list returned by the algorithm. For example, Rank-1 refers to the first hit of the search target, and Rank-5 refers to the possibility of the search target appearing in the first five matching targets. The advantage of the CMC curve is that it provides an intuitive understanding of the performance of the model, especially when considering the first few matching cases. However, the CMC curve cannot be applied to image retrieval with multiple cameras. Therefore, Zheng et al. [31] proposed using the mAP to evaluate the retrieval performance with multiple cameras. The mAP considers the average recall rate of all queries by first calculating the accuracy of each query and then averaging these accuracies to comprehensively reflect the performance of the model.

### 4.2. Experimental Settings

The experiment was implemented on a machine with an NVIDIA TITAN V GPU using the PyTorch deep learning framework version 1.4.0. The backbone network used was ResNet50 [25], and the last fully connected layer and average pooling layer were removed. The input image underwent preprocessing operations such as random horizontal flipping and cropping, and the image size was adjusted to 256×128. After normalization, it was input into the network model. The initial learning rate was 0.05, and after training for 30 epochs, the learning rate decayed to 1/10 of the original. A total of 60 epochs were trained; the final convolution stride was set to 2, and the batch size was set to 32. The hyperparameter *m* was set to 0.4, and *s* was set to 135. A Stochastic Gradient Descent (SGD) [32] optimizer was used for gradient updates, with weight decay set to $5\times 10^{-4}$ and momentum set to 0.9.

### 4.3. Comparison with Existing Methods

We compared the proposed person re-identification method with four existing person re-identification methods, and the results are shown in Table 2. Among them, the singular value decomposition network [33] (SVDNet) and pedestrian alignment network [34] (PAN) are person re-identification methods based on global feature learning which utilize dimensionality reduction, the orthogonalization of fully connected layers, and character alignment, respectively. The pose-driven deep convolutional (PDC) model [35] and attention-aware compositional network [36] (AACN) are person re-identification methods based on global and local feature learning which utilize pose estimation and alignment. Furthermore, DyReLU is short for Dynamic ReLU, as is the case below. It can be seen that the proposed person re-identification method has significantly improved performance on mainstream person re-identification datasets, indicating that the proposed method can obtain more robust pedestrian expression features in the feature extraction stage and can output more discriminative pedestrian features in the similarity measurement stage.

### 4.4. Ablation Study

#### 4.4.1. Incorporating Normalization-Based Channel Attention Module

From Table 3, it can be seen that incorporating the Normalization-based Channel Attention Module into the ResNet50 network improved the performance of the network model on both datasets. Among them, the improvement is more significant on the Market1501 dataset, which may be related to differences in the dataset itself. On the Market1501 dataset, the Rank-1 hit rate increased by 1.07%, and the mAP increased by 1.18%. On the DukeMTMC-ReID dataset, the Rank-1 hit rate increased by 1.21%, and the mAP increased by 0.93%.

#### 4.4.2. Using Dynamic ReLU

From Table 4, it can be seen that the use of dynamic activation functions slightly improved the performance of the network model on both datasets. Among them, on the Market1501 dataset, the Rank-1 hit rate increased by 0.04%, and the mAP increased by 0.73%. On the DukeMTMC-ReID dataset, the Rank-1 hit rate increased by 0.94%, and the mAP increased by 0.82%.

#### 4.4.3. Introducing Arcface Loss

From Table 5, it can be seen that introducing Arcface loss into cross-entropy loss significantly improved the performance of the network model on both datasets. On the Market1501 dataset, the Rank-1 hit rate increased by 0.77%, and the mAP increased by 1.43%. On the DukeMTMC-ReID dataset, the Rank-1 hit rate increased by 0.78%, and the mAP increased by 1.49%.

#### 4.4.4. Ablation Experiments to Verify the Effectiveness of Three Modules

To verify the effectiveness of various parts of the network model, we conducted ablation experiments on the Market1501 and DukeMTMC-ReID datasets. The Rank-1 and mAP obtained from each group of experiments are shown in Table 6. It can be seen that the proposed person re-identification method, which simultaneously introduces the NAM, dynamic activation function, and Arcface loss, performs the best on the Market1501 and DukeMTMC-ReID datasets, with Rank-1 improving by 1.28% and 1.4%, respectively; the MAP increased by 1.93% and 1.84%, respectively.

#### 4.4.5. Ablation Experiments to Determine the Values of Hyperparameters *m* and *s*

**Figure 8:**
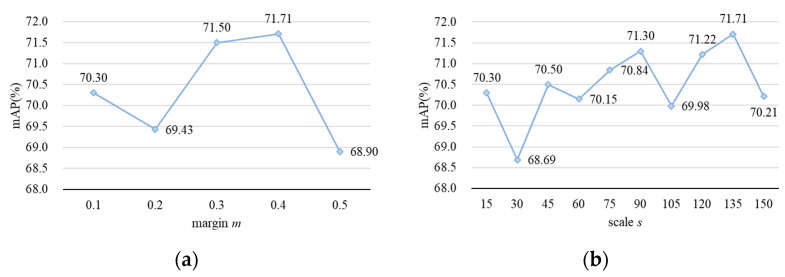
Results of ablation experiments on Arcface loss hyperparameters *m* and *s*. (**a**) The variation in mAP with different values of *m* while keeping the value of *s* equal to 135; (**b**) the variation in mAP with different values of *s* while keeping the value of *m* equal to 0.4.

Setting the margin (*m*) and scale (*s*) hyperparameters correctly is crucial for the effectiveness of Arcface loss. In order to achieve its best performance, we conducted ablation experiments on hyperparameters *m* and *s* on the Market1501 dataset. The Rank-1 and mAP obtained from each group of experiments are shown in Table 7 and Figure 8. It can be seen that for the proposed person re-identification method, the best performance is achieved when *m* and *s* are set to 0.4 and 135, with a 0.77% increase in the Rank-1 hit rate and a 1.43% increase in the mAP.

### 4.5. Visualization Analysis

**Figure 9:**
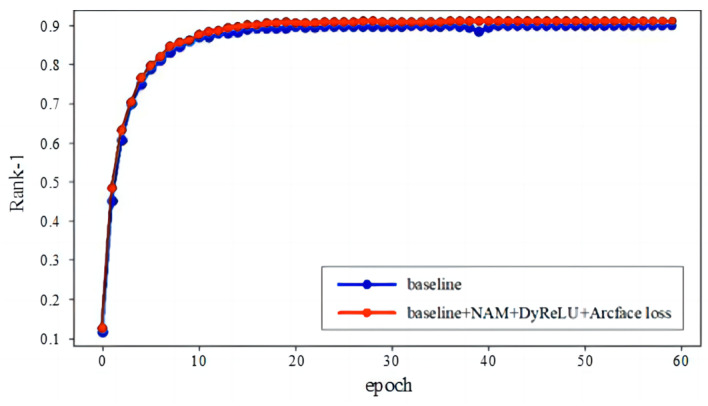
CMC evaluation curves of previous and proposed methods on Market1501 dataset.

The CMC evaluation curve of the proposed person re-identification method on the Market1501 dataset is shown in Figure 9. It can be seen that by incorporating Normalization-based Channel Attention Modules into the backbone network ResNet50, using dynamic activation functions for activation, and introducing Arcface loss into cross-entropy loss, the network model can perform better on the dataset. This indicates that the proposed method significantly improves expression ability in terms of pedestrian features and has stronger feature discrimination ability.

**Figure 10:**
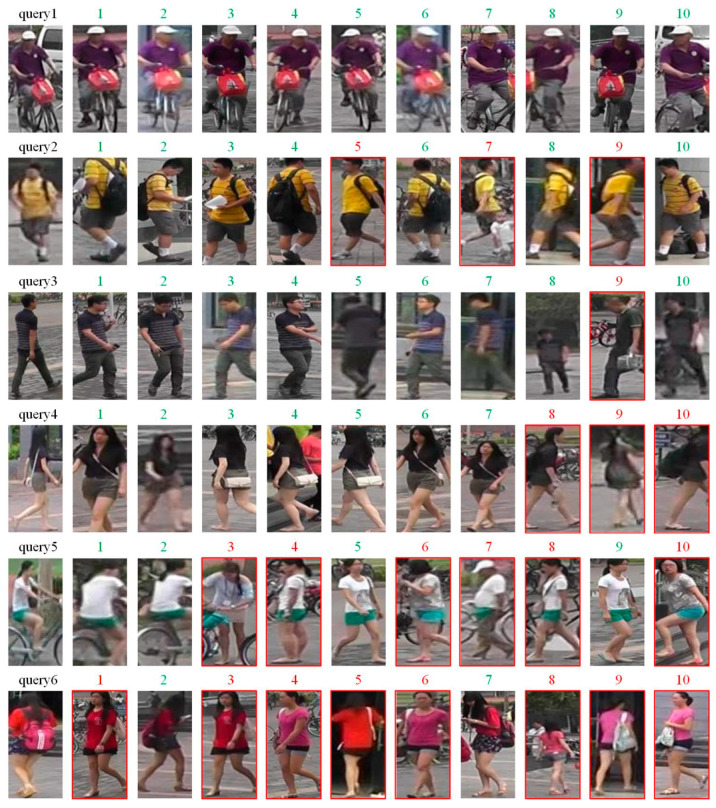
Visualization of person re-identification results using the proposed method.

We tested the proposed person re-identification method on the Market1501 dataset. The query results returned by the method are shown in Figure 10. Among them, query is the image to be queried, and 1–10 is the query result returned from the gallery library. The image with a green superscript is a positive sample image, which means it belongs to the same pedestrian entity as the query image to be queried. The image with a red superscript is a negative sample image, which means it does not belong to the same pedestrian entity as the query image.

From the results in Figure 10, it can be seen that for simple frontal facial images (query 1 and query 2), the proposed person re-identification method successfully extracts rich information for feature description and recognition. For more challenging samples, such as images of people from the back (query 3 and query 4), the proposed person re-identification method can also distinguish and recognize them to a certain extent in all visually similar images. Moreover, for a few pedestrian images to be queried (query 5 and query 6), the proposed person re-identification method cannot achieve ideal recognition results, which may be due to its insufficient ability to capture small differences such as clothing changes and pedestrian postures by global features.

## 5. Conclusions

We propose a novel person re-identification method based on global feature learning. Firstly, we incorporated the Normalization-based Channel Attention Module into the backbone network ResNet50 to selectively obtain more critical image information and output more representative pedestrian expression features. Secondly, we applied the dynamic activation function to the activation layer of the backbone network ResNet50, dynamically adjusting the activation method based on input, which enhanced the nonlinear expression ability of the network model for pedestrian features. Finally, we introduced Arcface loss into cross-entropy loss, which increased the inter-class distance while reducing the intra-class distance in angle space, making the network model output more discriminative regarding pedestrian features. In the experimental stage, we evaluated the enhanced model on two popular datasets, Market1501 and DukeMTMC-ReID, and used the mean average precision (mAP) and cumulative match characteristic (CMC) as performance evaluation metrics to comprehensively evaluate the proposed method. The results show that compared to other existing methods, the Rank-1 of the proposed method increased by at least 2.79% and 3.61%, respectively, and the mAP correspondingly increased by at least 5.34% and 2.13%. These results indicate that the model can extract more robust pedestrian features, enhance the distinguishability of features, and ultimately achieve higher recognition accuracy. In the future, we will explore how to further optimize algorithms, improve recognition accuracy, and provide more effective solutions to practical problems by introducing advanced object detection technologies to improve the receptive field and understanding ability of algorithms, or by adopting rich data augmentation strategies and advanced training methods to enhance the generalization ability and robustness of network models.

## Figures and Tables

**Table 1:** Datasets’ conditions.

Datasets	Year of Disclosure	Number of Cameras	Number of IDs	Number of Images
Market1501	2015	6	1501	32,668
DukeMTMC-ReID	2017	8	1404	36,411

**Table 2:** Performance comparison of different models on Market1501 and DukeMTMC-ReID datasets.

Method	Market1501		DukeMTMC-ReID	
	Rank-1	mAP	Rank-1	mAP
SVDNet [33]	82.30	62.10	76.70	56.80
PAN [34]	82.81	63.35	71.59	51.51
PDC [35]	84.14	63.41	-	-
AACN [36]	85.90	66.87	76.84	59.25
baseline	87.41	70.28	79.05	59.54
baseline + NAM + DyReLU + Arcface loss (ours)	88.69	72.21	80.45	61.38

**Table 3:** Performance comparison of models embedded with Normalization-based Channel Attention Module on Market1501 and DukeMTMC-ReID datasets.

Method	Market1501		DukeMTMC-ReID	
	Rank-1	mAP	Rank-1	mAP
baseline	87.41	70.28	79.05	59.54
baseline + NAM	88.48	71.46	80.26	60.47

**Table 4:** Performance comparison of models with Dynamic ReLU on Market1501 and DukeMTMC-ReID datasets.

Method	Market1501		DukeMTMC-ReID	
	Rank-1	mAP	Rank-1	mAP
baseline	87.41	70.28	79.05	59.54
baseline + DyReLU	87.45	71.01	79.99	60.36

**Table 5:** Performance comparison of models with Arcface loss on Market1501 and DukeMTMC-ReID datasets.

Method	Market1501		DukeMTMC-ReID	
	Rank-1	mAP	Rank-1	mAP
baseline	87.41	70.28	79.05	59.54
baseline + Arcface loss	88.18	71.71	79.83	61.03

**Table 6:** Results of ablation experiments on Market1501 and DukeMTMC-ReID datasets.

Method	Market1501		DukeMTMC-ReID	
	Rank-1	mAP	Rank-1	mAP
baseline	87.41	70.28	79.05	59.54
baseline + NAM	88.48	71.46	80.26	60.47
baseline + DyReLU	87.45	71.01	79.99	60.36
baseline + Arcface loss	88.18	71.71	79.83	61.03
baseline + NAM + DyReLU	88.36	71.60	80.12	60.78
baseline + NAM + Arcface loss	88.33	71.50	79.93	61.10
baseline + DyReLU + Arcface loss	88.12	71.88	80.44	60.86
baseline + NAM + DyReLU + Arcface loss	88.69	72.21	80.45	61.38

**Table 7:** Ablation experimental results for the hyperparameters *m* and *s* of Arcface loss on Market1501 dataset.

	*s* = 135	Rank-1	mAP		*m* = 0.4	Rank-1	mAP		*m* = 0.4	Rank-1	mAP
*m*				*s*				*s*			
0.1		87.41	70.30	15		87.26	70.30	90		88.12	71.30
0.2		86.37	69.43	30		86.28	68.69	105		86.73	69.98
0.3		87.80	71.50	45		87.14	70.50	120		88.12	71.22
0.4		88.18	71.71	60		87.50	70.15	135		88.18	71.71
0.5		87.23	68.90	75		87.95	70.84	150		87.83	70.21

## Data Availability

The data that support the findings of this study are available online. These datasets were derived from the following public resources: [Market1501, DukeMTMC-ReID].
